# A robust and miniaturized screening platform to study natural products affecting metabolism and survival in *Caenorhabditis elegans*

**DOI:** 10.1038/s41598-020-69186-6

**Published:** 2020-07-23

**Authors:** Julia Zwirchmayr, Benjamin Kirchweger, Theresa Lehner, Ammar Tahir, Dagmar Pretsch, Judith M. Rollinger

**Affiliations:** Department of Pharmacognosy, Faculty of Life Sciences, University of Vienna, Althanstraße 14, 1090 Vienna, Austria

**Keywords:** Drug discovery, Phenotypic screening, Caenorhabditis elegans, Natural products

## Abstract

In this study a robust, whole organism screening based on *Caenorhabditis elegans* is presented for the discovery of natural products (NP) with beneficial effects against obesity and age-related diseases. Several parameters of the elaborated workflow were optimized to be adapted for probing multicomponent mixtures combining knowledge from traditional medicine and NP chemistry by generating optimized small-scale extracts considering scarcity of the natural source, solubility issues, and potential assay interferences. The established miniaturized assay protocol allows for in vivo probing of small amounts of even complex samples (~ 1 mg) to test their ability to increase the nematodes’ survival time and the suppression of fat accumulation assessed by Nile red staining as hall marks of “healthy aging”. The workflow was applied on 24 herbal and fungal materials traditionally used against symptoms of the metabolic syndrome and revealed promising results for the extracts of *Gardenia jasminoides* fruits and the sclerotia from *Inonotus obliquus*. Tested at 100 µg/mL they were able to significantly reduce the Nile red fluorescence and extend the 50% survival rate (DT_50_) compared to the control groups. This phenotype-directed in vivo approach opens up new horizons for the selection of natural starting materials and the investigation of their active principles as fast drug discovery tool with predictive value for human diseases.

## Introduction

**Figure 1:**
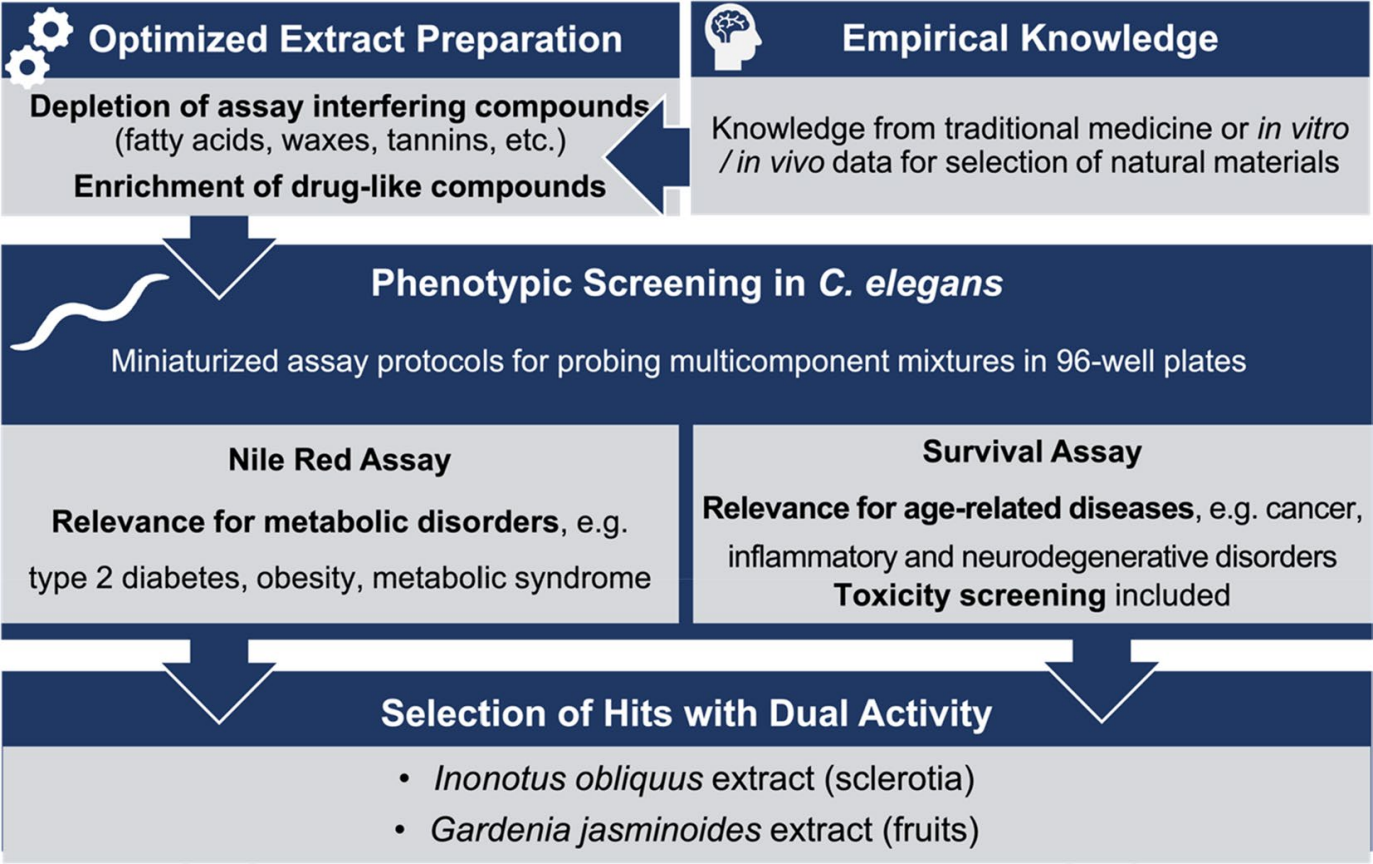
Workflow of the presented approach.

*Caenorhabditis elegans* (Maupas, 1900), a 1 mm sized roundworm, is a popular model organism in almost all areas of modern biology. It can be maintained at low cost, has a short reproductive cycle of three days with a large brood size of 300 progenies per hermaphrodite worm and a transparent body comprising exactly 959 somatic cells^1^. In recent years it has been increasingly used as a model organism for drug screenings^2–7^. The fundamental idea behind is that basic molecular processes which are causal for the development of diseases including aging processes are conserved in the animal kingdom. Indeed *C. elegans* shares many similarities with humans such as autophagy, mitochondrial regulation, apoptosis, proteostasis, energy control, fat-storage, stress response systems and neuronal regeneration^8–16^. A recent meta-analysis estimated that 41.7% of the protein-coding genes in *C. elegans* have orthologs in humans^17^. In this light, screening for substances beneficial to a disease phenotype in *C. elegans* can have important predictive value also for human diseases^18, 19^. The simplicity and tractability of the worm compared to classical mammal models represents a large advantage. Its small size makes it amenable to whole organism screening in microtiter plates for medium/high-throughput screening with little consumption of materials and sample^20^. This possibility is particularly helpful for drug discovery from natural sources, which is often impeded by scarcity of natural starting materials, and even more relevant for their isolates which require tedious isolation or synthesizing efforts^21–24^. There is an increasing interest in these approaches as shown by a growing number of isolated NP and extract screens^25–30^. However, extracts are complex mixtures consisting of a broad range of metabolites with different chemical properties which are not necessarily drug-like. Some are prone to give false-positive or false-negative results due to insolubility or interferences with optical readouts^31–34^. Therefore, in this study special attention was given to an extract preparation which allows the enrichment of lead-like constituents and meets the requirements for a reliable readout in the phenotypic *C. elegans* assays. By means of these optimized extracts a screening protocol was established with two robust and miniaturized *C. elegans*-based assays for performing survival and Nile red fluorescence experiments capable of testing multicomponent mixtures and their isolates (Fig. 1). Natural products exerting a significant reduction in the *C. elegans*-based Nile red assay avoid of any lifespan shortening or even additionally causing an increase in lifespan have been identified. They are considered as promising candidates in the search for NP able to overcome metabolic diseases as well as age-related diseases with a prescreened low risk of toxicity.

## Results and discussion

### Selection of natural raw materials

Selection of starting materials was guided by literature on NPs with potential effects on human health. The following criteria for the selection of the starting material were considered:

(i) a species (and its corresponding organ) traditionally used in the field of metabolic disorders, enhancement of the immune system and promotion of health and longevity, and/or
(ii) in vitro data from extracts derived from enzyme or cell-based assays which lack more in-depth analysis in an in vivo model organism, and/or
(iii) pharmacological in vivo data of NPs with effects related to metabolic syndrome and/or anti-aging effects.

The selection presented in this study comprises 24 materials from plant and fungal species belonging to 20 different families, either used in Traditional Chinese, European, African or American medicines, but also in different medicinal systems such as e.g. Ayurveda, Unani or Siddha systems. The literature results for the selected 24 species is given in Table S1.

### Survival assay

An increase in lifespan is considered to be a result of interventions in pathways of aging. The measurement of lifespan is therefore a key experiment leveraged to seek for lifespan delaying agents endowed with the potential to treat age-related diseases in vertebrates/humans^35,36^. This investigation requires analysis of survival over time during the aging process of an individual in a population. Survival assays in *C. elegans* have been performed in both, solid and liquid culture at temperatures ranging from 15 °C to 25 °C^36^. The use of liquid culture offers substantial benefits over solid culture: (1) nematodes encounter less mechanical stress when assayed in liquid culture^37^, since no prodding or transferring of worms with a platinum wire is necessary, (2) there is no need of censoring worms that are buried into the solid media, (3) it is adaptable to large-scale screenings^38^, and (4) less sample amount is needed. In this study, survival analyses were performed at 25 °C with N2 wild-type worms and the commonly used liquid medium S-complete supplemented with OP50 bacteria. The survival of worms at 25 °C was recorded three times a week until only 50% of the worms were alive (death time 50%; DT_50_). DT_50_ for survival measurement was chosen because it enables a shorter observation time, compared to standard protocols with the necessity of survival monitoring until death of all individuals within a population. Death was determined when the worms did not move after 2 min shaking on a microplate-shaker or exposure to strong light. Many parameters influencing the nematodes‘ survival and well-being such as the population density and feeding frequency were adapted based on previous findings^39^.

### Nile red assay

Triacylglycerol stores can be stained with the solvatochromatic dye Nile red mixed into the worms’ bacterial OP50 diet. In aqueous medium Nile red is almost non-fluorescent. By mixing it with OP50 bacteria and upon feeding, it accumulates in the nematodes’ subcellular compartments, the so-called lysosome related organelles. In this environment rich in polar lipids Nile red exposes a strong red to yellow-shifted fluorescence^40–42^. The intensity of this fluorescence has been shown to correlate with the worms’ fat content, why this method has been used for chemical and genetic screens for small molecules and genes that affect fat metabolism^11,43–46^. Compared with biochemical and chromatographic methods the vital Nile red assay is more suitable for rapid large-scale screenings in a miniaturized format and can be performed in S-complete medium filled in 96-well plates. *C. elegans* mutant strain SS104, bearing a temperature-sensitive mutation in valyl aminoacyl tRNA synthetase *glp-4(bn2ts)*, was employed for screening at the restrictive temperature of 25 ± 0.5 °C*. Glp-4(bn2ts)* mutant worms develop normal at 16 °C but are sterile at the experimental temperature of 25 °C^47,48^*.*

#### Optimization of treatment time

For the determination of an ideal treatment time L4 worms were incubated at 25 °C for up to 7 days, and Nile red fluorescence was measured each day. An increase of fluorescence was observed from day 2 to 6 followed by a decline (Fig. S1). This finding is in accordance with results of Shen et al.^49^, who determined an increased fat accumulation of N2 worms from day 2 to 6 measured with biochemical methods. However, for chemical screens not only the time of fat accumulation itself but also the accumulation and the persistence of chemicals in the worms have to be considered. It has been shown that drug concentrations in worms gradually decrease after the first day of treatment^50^. Based on these findings and our observations we selected a treatment of 5 days starting from L4 staged worms. This time point guaranteed a high Nile red fluorescence indicating a sufficient fat accumulation as well as persistence of chemicals in worms. Furthermore, an additional feeding within these 5 days was not necessary.

### Optimization of DMSO concentration

**Figure 2:**
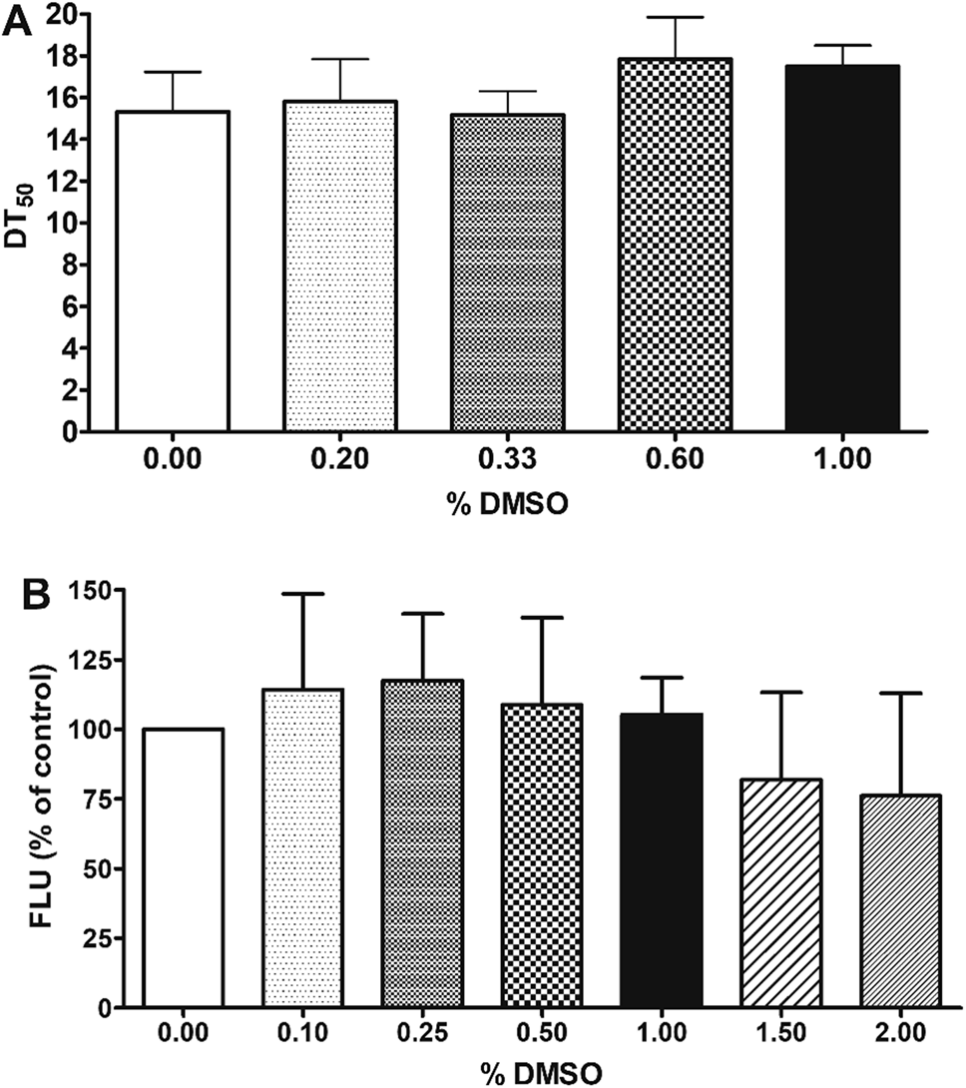
Effect of different DMSO concentrations (0.00—2.00% in S-complete medium) on (**A**) the survival and (**B**) vital Nile red staining of *C. elegans*; (**A**) Bars represent the mean DT_50_s of N2 worms in days ± SD. Survival assay tested in parallel triplicates. (**B**) Bars represent the mean vital Nile red fluorescence intensities of SS104 worms of five independent experiments expressed as % of control worms (0% DMSO) ± SD.

Low solubility of NPs is a well-known issue^51^, but complete solubility as a prerequisite of bioavailability is a crucial requirement for testing bioactivity in a phenotypic-based assays^52,53^. Dimethyl sulfoxide (DMSO) guarantees chemical stability and dissolves samples with a wide range of chemical properties^54,55^. However, DMSO itself has shown to effect *C. elegans* phenotypes^56,57^. It was reported that a DMSO concentration higher than 0.60% shortens the lifespan of *C. elegans* in liquid culture and should be avoided in survival studies^39^. Other sources indicate that concentrations between 0.80 – 1.00% DMSO lead to a significant increase in median lifespan (by 15%) when using DMSO from egg-stage throughout life, although these effects did not occur when worms were treated after egg-laying period^57^. To find the optimal concentration of DMSO for both the survival assay and Nile red assay, we tested different concentrations of DMSO and could observe a lifespan prolonging tendency (without significance) ranging from 0.00 to 1.00% of DMSO treatment (Fig. 2A). The mean DT_50_ value of untreated worms in S-complete medium was 15.33 days compared to 15.83, 15.17, 17.83 and 17.50 days for worms treated with DMSO concentrations of 0.20%, 0.33%, 0.60% and 1.00%, respectively (Table S2). Those results are consistent with the study of Wang et al.^57^, who has shown that nematode growth media (NGM) plates containing DMSO concentrations from 0.01% to 2.00% led to a dose-dependent lifespan extension, reaching its maximum at 0.5%. The effect of DMSO on Nile red staining of SS104 nematodes was minor when tested up to concentrations of 1.00% with a mean fluorescence of 105.30 ± 13.28% compared to untreated worms. The two highest tested concentrations, 1.50 and 2.00% DMSO, caused an insignificant reduction of fluorescence (Fig. 2B). Thus, it can be concluded that 1.00% DMSO can be used for chemical screens without significantly influencing the nematodes’ survival or Nile red fluorescence.

### Positive controls

#### Survival assay

**Figure 3:**
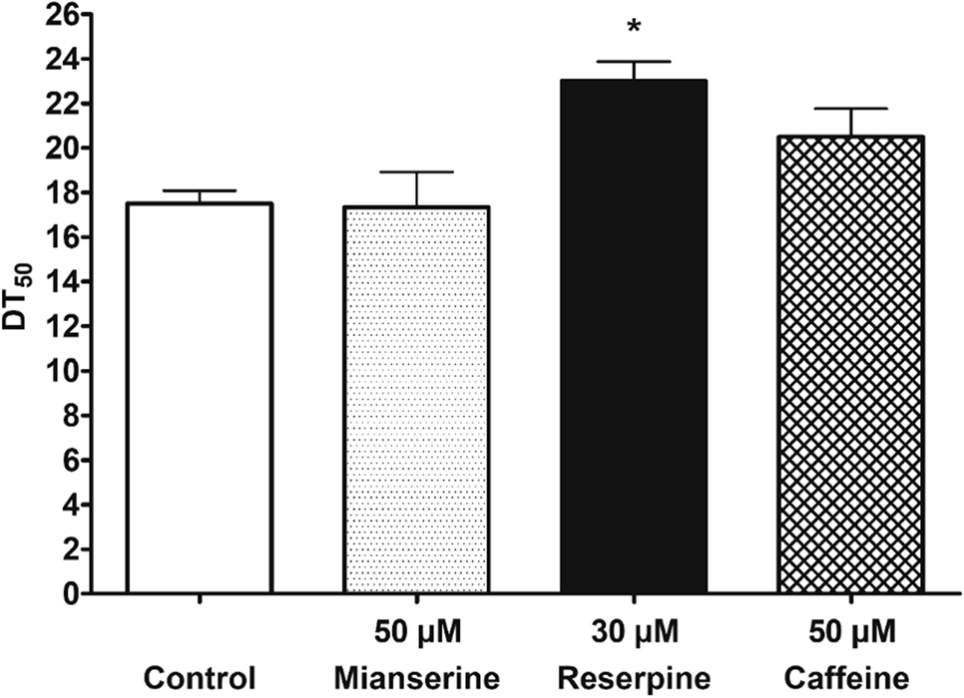
Screening of positive controls. Bar charts of mean DT_50_ values of *C. elegans* treated with mianserine (50 µM), reserpine (30 µM) and caffeine (50 µM) in comparison to vehicle control. Reserpine at 30 µM significantly extended the DT_50_ of wild type *C. elegans* by 31.4%. Bars represent the DT_50_ value in comparison to the control group ± SD of three parallel experiments. Significance was assessed by one way ANOVA with Dunnett post-test (*p < 0.05).

A number of different drug substances have been reported to prolong the nematodes’ lifespan (e.g. the antidepressant mianserine has previously been shown to extend the nematodes’ lifespan by 31% when given throughout adult life at a concentration of 50 µM^2^). Interestingly, this effect was only present at 20 °C and abolished when the survival assay was performed at 25 °C^58^. The anti-hypertensive drug reserpine has been reported to increase *C. elegans* lifespan by 31% and 64% when worms were treated with 30 µM reserpine from embryo stage onwards and from young adult stage on, respectively. This effect was also temperature dependent and only present at 25 °C. No significant lifespan extension was observed at 20 °C^59^. Further, the xanthine caffeine has been reported for its lifespan extending potential when nematodes were exposed to various concentrations of caffeine ranging from 10 to 200 µg/mL at 20 °C^60^ (corresponding to 51.5–1,000 µM). For validation of the presented assay conditions, mianserine 50 µM, reserpine 30 µM and caffeine 50 µM were tested (Fig. 3). In our assay settings, no significant DT_50_ extension was observed for the antidepressant mianserine in comparison to the control group; for the natural stimulant caffeine a moderate DT_50_ extension was recorded. In line with the previously mentioned data, reserpine at 30 µM resulted in a significant DT_50_ extension of 31.43% (*p* < 0.05, Table S3) and was henceforth used as positive control in all lifespan experiments.

#### Nile red assay

**Figure 4:**
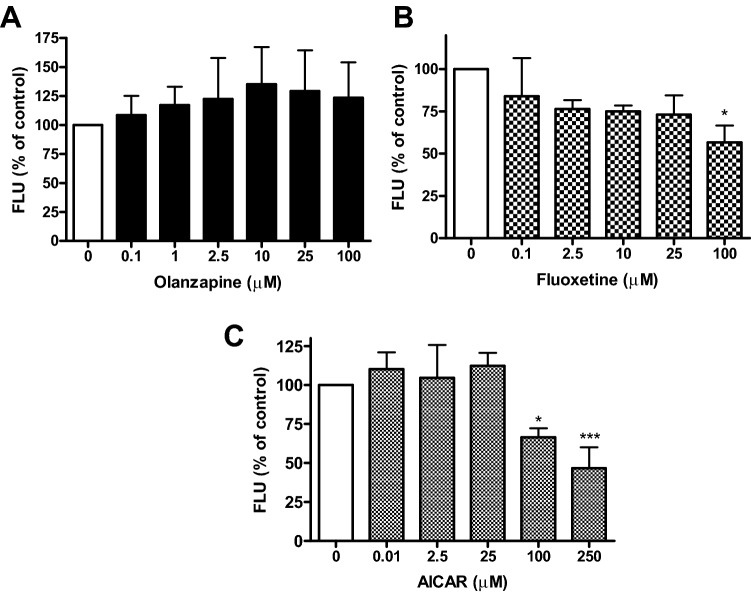
Effects of drugs on Nile red fluorescence of SS104 *C. elegans*. Bar charts represent the mean Nile red fluorescence of three independent experiments (± SD). Worms were treated with different concentrations of (**A**) olanzapine, (**B**) fluoxetine, and (**C**) AICAR normalized to vehicle treated control worms. Statistical significance assessed by one way ANOVA and Bonferroni post-test (*p < 0.05, ***p < 0.001).

For assay validation we tested three pharmacological agents reported to influence mammal and *Caenorhabditis elegans* fat stores (Fig. 4) i.e. olanzapine, fluoxetine and 5-aminoimidazole-4-carboxamide-ribonucleoside (AICAR). Worms treated with olanzapine exhibited an increased Nile red fluorescence of 135.3% and 129.3% at 10 and 25 μM, respectively, compared to vehicle treated worms. On the contrary, fluoxetine showed a dose dependent fluorescence-reducing effect. At 100 μM fluoxetine significantly reduced the mean fluorescence to 56.7% compared to vehicle treated worms. AICAR similarly reduced Nile red fluorescence but at higher concentrations. Treatments of 250 and 100 μM AICAR significantly reduced the mean Nile red fluorescence to 46.7% and 66.4%, respectively, compared to vehicle treated worms. The response to the three compounds suggests that the *C. elegans* Nile red assay has a certain degree of translatability to mammals as all three tested pharmacological agents are known to modulate mammalian fat similar, i.e. olanzapine is known to exacerbate hyperlipidemia and type-2 diabetes and induce fat accumulation in humans^61–63^; the serotonin reuptake inhibitor fluoxetine is known to lead to weight reduction in obese patients^64^ and the investigational drug and AMPK activator AICAR blocked high-fat diet induced body weight gain in mice^65^.

### Extract preparation adapted to phenotypic screening

Although nature has been one of the most prolific resources for drug discovery^66^, the isolation of their bioactive compounds and their physicochemical characterization are often tedious. Screening of extracts is a useful approach for the selection of natural starting material worth a phytochemical work-up. However, the complexity^51,67^ of an organism’s metabolome and its matrix can account for profound issues when screened in biological systems. Assay perturbation, aggregating metabolites and dynamic residual complexities are prone to give false-positive or false-negative results^33^. The concept of front-loading an extract with drug- or lead-like properties was previously described by Camp et al.^67^. This process facilitates drug discovery from natural sources by enrichment of compounds that meet the physico-chemical properties of drug-like molecules. Additionally, the depletion of highly apolar constituents (e.g. waxes, lipids) and oligo- and polymeric polyphenols (e.g. tannins), not only significantly reduces problems arising during the extracts’ analysis and chromatographic work-up (e.g. clogging of columns), but also diminishes the risk of assay interferences^23^. Complex mixtures comprising compounds with very different molecular weights and polarity may have challenging implications when it comes to solubility, especially when handling a liquid assay^68^. Further, it has to be considered that minor bioactive constituents within multi-component mixtures may easily been overseen, and accordingly ask for assays with high sensitivity^33,52,68^. To integrate extracts to our liquid-based screening platform, we aimed to establish a standardized small-scale extraction procedure with an enriched yield of drug-like secondary metabolites and depleted primary metabolites and assay-interfering compounds. A detailed extraction procedure is provided in the Supplementary Information (Protocol for the preparation of optimized small-scale extracts). Small-scale dichloromethane (CH_2_Cl_2_) and methanol (MeOH) extracts were generated as previously described^67,69,70^, with some alterations. Briefly, dried natural materials were ground and defatted with *n*-hexane twice. The remaining materials were successively extracted with CH_2_Cl_2_ and MeOH. Both extracts were combined. A depletion of potentially included tannins was performed with solid-phase extraction (SPE). These small-scale extracts optimized for their application in the miniaturized *C. elegans* assays were transferred to pre-weighted and labeled Eppendorf tubes, dried in a desiccator and the total extract weight was determined using an analytical balance. Aliquots of the transferred extract were dissolved in DMSO to a concentration of 10 mg/mL and stored at − 20 °C until use.

### NPs applicability on established screening platform

**Figure 5:**
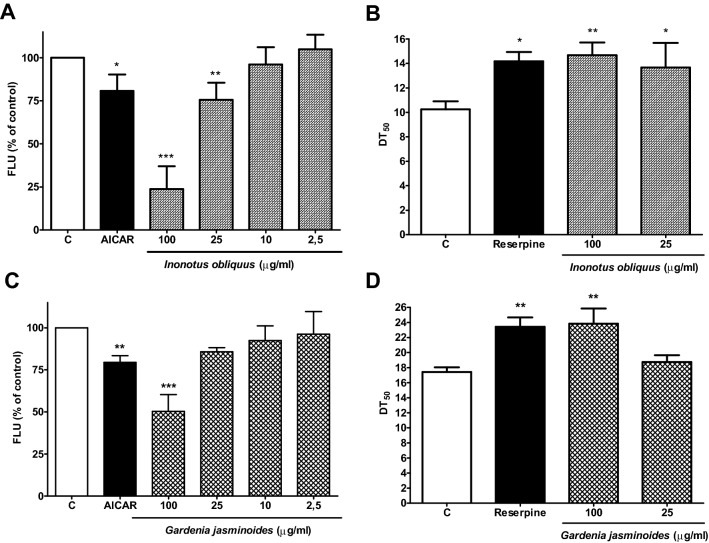
Effect of *I. obliquus* and *G. jasminoides* extracts on Nile red fluorescence (A, C) and survival (B, D) of *C. elegans.* Nile red assay (**A**, **C**): Vital Nile red fluorescence of *C. elegans* treated with control, AICAR (100 μM) and extracts of *I. obliquus* (**A**) and *G. jasminoides* (**C**). Bars represent the mean fluorescence intensities of at least three independent experiments expressed as % of control worms ± SD. Significance was assessed by One-Way ANOVA and Bonferroni post-test (***p < 0.001; **p < 0.01; *p < 0.05). Survival assay (**B**, **D**): Worms were treated with control, reserpine (30 µM) and extracts of *I. obliquus* (**B**) and *G. jasminoides* (**D**). Bars represent the mean DT_50_ ± SD of three parallel experiments. Significance was assessed by One-Way ANOVA and Dunnett’s post-test (*p < 0.05; **p < 0.01).

The phenotypic screening was exemplarily performed with 24 optimized small-scale extracts of herbal and fungal materials (Table 1), which have either been traditionally used against symptoms related to the metabolic syndrome or described to be active in any kind of animal model in this disease area. All extracts were tested at two concentrations (25 and 100 µg/mL) for their effect on survival and Nile red staining in our liquid-based *C. elegans* screening platform (Table S1). Six extracts exerted a significant reduction of Nile red fluorescence (i.e*. Inonotus obliquus*, *Terminalia chebula*, *Valeriana officinalis*, *Imperata cyclindrica*, *Gardenia jasminoides* and *Scutellaria barbata*), and nine extracts showed a pronounced increase in the nematodes’ lifespan; the 50% survival rate of wild-type worms was significantly extended by six small-scale extracts tested at 100 µg/mL (i.e. *Gloeophyllum odoratum*, *Pimenta dioica*, *I. obliquus*, *Syzygium aromaticum*, *Cynanchum stauntonii* and *G. jasminoides*) and one extract even at 25 µg/mL (i.e. *Cynanchum paniculatum*). Two extracts, i.e. those of *Euphrasia officinalis* herbs and *Eriobotrya japoinica* leaves were capable of significantly increasing the 50% survival rate at both tested concentrations. On the contrary, the root extract of *Peucedanum ostruthium* drastically decreased the nematodes’ DT_50_ at both test concentrations. Correspondingly, this extract was not evaluable at 100 µg/mL in the Nile red assay, since most worms were already dead. From all the tested samples only two extracts significantly reduced the worms’ Nile red fluorescence at both tested concentrations, 25 and 100 µg/mL (i.e. *I. obliquus* and *I. cyclindrica*). The reduction of Nile red fluorescence by the extract of *V. officinalis* was however accompanied by a decreased worm survival. *T. chebula*, *I. cyclindrica* and *S. barbata* decreased Nile red fluorescence and increased (although not significantly) survival of worms. Intriguingly, the extracts of *I. obliquus* and *G. jasminoides* showed both, a significantly increased survival rate and reduced Nile red fluorescence (Fig. 5).

**Table 1:** Plant and mushroom materials for extraction.

*Species*	Family	Organ	Source/sample location	Voucher specimen/charge number
*Andrographis paniculata* (Burm.f.) Nees	Acanthaceae	herb	Plantasia GmbH, Oberndorf bei Salzburg, Austria	JR-20150313-A1 Ch.Nr.: 780,672
*Azadirachta indica* A.Juss	Meliaceae	fruits	Padma AG, Wetzikon, Switzerland	JR-20150615-A1 Ch.Nr.: 2,021,108,301
*Calendula officinalis* L.	Asteraceae	flowers	Padma AG, Wetzikon, Switzerland	JR-20150615-A9 Ch.Nr.: 21,348,300
*Cetraria islandica* (L.) Ach	Parmeliaceae	lichen	Padma AG, Wetzikon, Switzerland	JR-20150615-A7 Ch.Nr.: 20,885,300
*Cynanchum paniculatum* (Bunge) Kitag. ex H.Hara	Apocynaceae	roots	Plantasia GmbH, Oberndorf bei Salzburg, Austria	JR-20150313-A2 Ch.Nr.: 840,274
*Cynanchum stauntonii* (Decne.) Schltr. ex H.Lév	Apocynaceae	rhizomes	Plantasia GmbH, Oberndorf bei Salzburg, Austria	JR-20101012-A1 Ch.Nr.: 310,051
*Drynaria fortunei* (Kunze ex Mett.) J.Sm	Polypodiaceae	rhizomes	Plantasia GmbH, Oberndorf bei Salzburg, Austria	JR-20150313-A3 Ch.Nr.: 030,161
*Eriobotrya japoinica* (Thunb.) Lindl	Rosaceae	leaves	Plantasia GmbH, Oberndorf bei Salzburg, Austria	JR-20110421-A1 Ch.Nr.: 030,724
*Euphrasia officinalis* L.	Orobanchaceae	herb	Kottas Pharma GmbH, Vienna, Austria	JR-20090625-A1 Ch.Nr.: KLA90309
*Fomitopsis pinicola* (Sw.) P. Karst. (strain 10)	Fomitopsidaceae	fruit body	Viggartal, Ellbögen, Austria (grown on dead spruce trunk); Ursula Peintner	FompinE0010
*Ganoderma lucidum* (Curtis) P. Karst	Ganodermataceae	fruit body	Plantasia GmbH, Oberndorf bei Salzburg, Austria	JR-20150313-B1 Ch.Nr.: 680,898
*Gardenia jasminoides* J.Ellis	Rubiaceae	fruits	Plantasia GmbH, Oberndorf bei Salzburg, Austria	JR-20150313-A1 Ch.Nr.: 11 0,284
*Gloeophyllum odoratum* (Wulfen) Imazeki	Gloeophyllaceae	fruit body	Oberperfuss, Austria (grown on spruce) Ursula Peintner	JR-20140310-A1 GloodoE0054
*Imperata cylindrica* (Nees) C.E.Hubb	Poaceae	rhizomes	Plantasia GmbH, Oberndorf bei Salzburg, Austria	JR-20150313-A4 Ch.Nr.: 81 0,178
*Inonotus obliquus* (Ach. ex Pers.) Pilát	Hymenochaetales	sclerotia	Finnland, 1998 Ursula Peintner	UP-20121212-A1
*Peucedanum ostruthium* (L.) W.D.J.Koch	Apiaceae	roots /rhizomes	Kottas Pharma GmbH, Vienna, Austria	JR-20180119-A2 Ch.Nr.: P17301770
*Pimenta dioica* (L.) Merr	Myrtaceae	fruits	Padma AG, Wetzikon, Switzerland	JR-20150615-A4 Ch.Nr.: 21,362,100
*Piptoporus betulinus* (Bull.) P. Karst. (strain 39)	Fomitopsidaceae	fruit body	Vahrn bei Brixen, Italy, grown on birch, Ursula Peintner	PipbetE0039
*Potentilla aurea* L.	Rosaceae	herb	Padma AG, Wetzikon, Switzerland	JR-20150615-A3 Ch.Nr.: 21,161,301
*Scutellaria barbata* D.Don	Lamiaceae	herb	Plantasia GmbH, Oberndorf bei Salzburg, Austria	JR-20150313-A5 Ch.Nr.: 76 0,577
*Sida cordifolia* L.	Malvaceae	herb	Padma AG, Wetzikon, Switzerland	JR-20150615-A2 Ch.Nr.: 20,981,300
*Syzygium aromaticum* (L.) Merr. & L.M.Perry	Myrtaceae	flowers	Padma AG, Wetzikon, Switzerland	JR-20150615-A8 Ch.Nr.: 21,321,101
*Terminalia chebulia* Retz	Combretaceae	fruits	Padma AG, Wetzikon, Switzerland	JR-20150615-A5 Ch.Nr.: 21,324,301
*Valeriana officinalis* L.	Caprifoliaceae	roots	Padma AG, Wetzikon, Switzerland	JR-20150615-A6 Ch.Nr.: 21,388,100

*Inonotus obliquus* is a parasitic mushroom belonging to the Hymenochetaceae family. Its sclerotia, commonly known as chaga conks, grow on hardwood trees. Chaga decoctions are traditionally used in Eastern Europe and Russia for different therapeutic indications such as cancer, inflammation and metabolic disease^71,72^. In our screening, the extract (HPLC chromatogram Fig. S2) significantly inhibited the Nile red fluorescence at 100 and 25 μg/mL and extended the mean survival rate of worms with a significant DT_50_ increase of 43.1% and 33.3%, at 100 and 25 µg/mL, respectively (Table 2). Our findings are in line and complement recent preclinical studies on the beneficial effects of *I. obliquus* extracts in different mammal models of obesity and diabetes^73–76^. Longevity effects *of I. obliquus* in *C. elegans* were already reported by Scerbak et al.^77,78^, and in *Drosophila melanogaster* by Zhang et al.^79^.

**Table 2:** Survival analysis upon treatment with *I. obliquus.*

		Mean DT_50_ ± SD	*N*	DT_50_ extension (%)	*p*-value
Control		10.33 ± 0.66	220	–	
Reserpine	30 µM	14.17 ± 0.76	138	38.21	*p* < 0.05
*I. obliquus*	100 µg/mL	14.67 ± 1.04	119	43.09	*p* < 0.01
*I. obliquus*	25 µg/mL	13.67 ± 2.02	112	33.33	*p* < 0.05

*N* is the total number of worms assayed for survival. One way ANOVA with Dunnett’s post-test was used for statistical evaluation. *P*-value < 0.05 was considered as statistically significant.

*Gardenia jasminoides* is an evergreen shrub belonging to the Rubiaceae family. Its fruits are traditionally used in China because of their diuretic, cholagogue, anti-inflammatory and hypoglycemic properties^80,81^. The extract (HPLC chromatogram Fig. S3) significantly increased the 50% survival rate of N2 worms by 36.84% (Table 3) and inhibited Nile red fluorescence at 100 μg/mL. *G. jasminoides* and its isolated constituents e.g. geniposide, genipin, crocetin and crocin are already known to have anti-obesity, hypolipidemic and hypoglycemic effects in different mammal models^82–84^. Although some beneficial effects of genipin/geniposide on ageing such as cognition in aged fruit-flies and age-related insulin resistance in rats are reported^85,86^, it is the first report of a longevity promoting effect described for the extract of *G. jasminoides* fruits.

## Conclusion

The preclinical evaluation of efficacy of multicomponent mixtures is a crucial step in drug discovery from nature. For the selection of the herein probed NPs, we decided to consider the ethnopharmacological relevance, since such samples are supposed to be more likely active^87,88^ and—proven by a long-standing application—they are considered to be safer and in turn less toxic^89^ compared to samples from randomly collected species. Starting from crude extracts it might be a tedious and costly process often obstructed by many pitfalls due to the presence of highly abundant bulk substances (e.g. lipids, waxes, chlorophyll, tannins) that are commonly known to interfere with biological assays^24^. With this study, we emphasize the need to perform a thoroughly performed phyto-/mycochemical extraction procedure that allows the separation of assay interfering compounds and facilitates the enrichment of putatively bioactive metabolites in a multicomponent mixture. The herein presented 96-well plate liquid format enables a robust, time- and resource-efficient, thus cost-effective screening of extracts. For the presented survival assay (counting up to one month) roughly 240 samples (including controls) can be managed by one operator (evaluated by three wells in parallel). For the Nile red assay, in one month about 400 samples (including controls) are manageable per operator (evaluated by six wells in parallel). These numbers refer to a setup without any technically challenging devices (except for a fluorescence microscope). With more sophisticated and costly instrumentation the herein presented *C. elegans* assays are adaptable to automatization for screenings of larger collections^20^. These collections could also include samples from less researched resources, e.g. marine organisms, terrestrial microbes or randomly selected plants and fungi^90^.

Several parameters have been optimized in this study to overcome challenges usually posed by assaying multi-component mixtures in a phenotypic screening^34^. *C. elegans* is an established living model for obesity and aging research^49^. Adapted for probing of extracts in miniaturized form (on 96-well plates) this phenotypic screening workflow serves as valuable preclinical tool using even small quantities of multicomponent mixtures (less than 1 mg) to assess their potential to interfere in lipid metabolism and age-related diseases. A further big advantage is the concurrent toxicity prescreening in the *C. elegans* lifespan assay^37^. Intriguingly, in our experiments only one out of 24 extracts showed a significant nematotoxicity, namely the root extract of *Peucedanum ostruthium.* The nematodes’ DT_50_ value was decreased by roughly 45% and 60% at 25 and 100 µg/mL, respectively, when compared to the untreated control.

Although the observed efficacy in *C. elegans* has to be critically validated to stand the test also in higher animal models, this workflow enables the rapid identification of auspicious extracts for further chemical, pharmacological and toxicological investigations. The tractability of *C. elegans* to genetic manipulations and the availability of genetic and biochemical tools further facilitates a straight-forward identification of involved pathways and genes^91,92^.

The screening applied with optimized extracts (tested at 25 and 100 µg/mL) from 24 herbal/fungal drugs underlines the suitability of the generated workflow. The data revealed a considerable amount of extracts significantly increasing the worms’ lifespan (9 out of 24 at least at one of the tested concentrations) and decreasing the worms’ Nile red fluorescence (6 out of 24 at least at one of the tested concentrations).

Two extracts even exerted both, a reduction in *C. elegans* Nile red fluorescence and an increase in the nematodes’ survival. The most pronounced dual activities were observed for the extracts of Chaga and *G. jasminoides* fruits, thus offering a superb starting point for future investigations in the search for natural products improving healthy aging and the discovery of natural lead structures able to conquer metabolic diseases and age-related pathologies.

**Table 3:** Survival analysis upon treatment with *G. jasminoides.*

		Mean DT_50_ ± SD	*N*	DT_50_ extension (%)	*p*-value
Control		17.42 ± 0.63	213	–	
Reserpine	30 µM	23.42 ± 1.23	208	34.45	*p* < 0.01
*G. jasminoides*	100 µg/mL	23.83 ± 2.02	97	36.84	*p* < 0.01
*G. jasminoides*	25 µg/mL	18.75 ± 0.90	88	7.66	ns

N is the total number of worms assayed for survival. One way ANOVA with Dunnett’s post-test was used for statistical evaluation. P-value < 0.05 was considered as statistically significant.

## Methods

### Natural materials

Voucher specimens are deposited in the Herbarium of the Department of Pharmacognosy, University of Vienna, Austria.

#### Generation of extracts

Optimized small-scale extracts were prepared as previously described in Kratz et al.^69^ adapted from Camp et al.^67^. In short, ~ 300 mg of dried, pulverized material were defatted twice with 5 mL *n*-hexane (AnalaR NORMAPUR ACS, ≥ 95%). The remaining material was extracted successively with 7 mL CH_2_Cl_2_ (GPR RECTAPUR, ≥ 99%) and 13 mL MeOH (AnalaR NORMAPUR ACS, ≥ 99.8%) at RT under sonication for 15 min. The extracts were filtered, combined and dried under vacuum. For tannin depletion, the dried CH_2_Cl_2_-MeOH extract was restored in 4 mL MeOH, loaded onto a 3 mL solid-phase extraction cartridge (phenomenex, AH0-7001) filled with polyamide gel (CC-6; 900 mg) and washed two times with MeOH. The tannin-free sample was dried again under vacuum to deliver the final extract. Dried small-scale extracts were dissolved in DMSO (Rotipuran ≥ 99.8%, p.a.) to a final concentration of 10 mg/mL. Samples were stored at -20 °C until used.

### *Caenorhabditis elegans* strains, maintenance and synchronization

*Caenorhabditis elegans* wild-type var. Bristol N2, the mutant SS104 *glp-4(bn2ts)*, and *E. coli* OP50 were obtained from the *Caenorhabditis* Genetics Center (University of Minnesota). OP50 were grown in LB medium for eight hours at 37 °C, harvested through centrifugation, washed with double distilled water (ddH_2_O) and were air-dried. Then bacteria were suspended in S-complete medium at a concentration of 100 mg/mL. Flasks were stored at 4 °C until use. Hermaphrodite animals were maintained on nematode growth medium (NGM) agar plates seeded with 200 µL of OP50 solution at 16 °C according to standard protocols^93^. For maintenance worms were transferred to new plates every week and cultures were monitored on a regular basis. For preparation of a synchronized worm population N2 worms were chunked three days, SS104 worms were chunked four days before synchronization. Synchronization was performed by bleaching technique^39,94,95^. Briefly, worms were washed of the plates with ddH_2_O and treated with bleaching solution for approximately 5–10 min. The lysis of the worms was controlled under a dissecting microscope. Then isolated eggs were pelleted and washed twice with M9 buffer and S-complete medium. Eggs were kept in S-complete medium for 42 h with gentle agitation and sufficient aeration until the synchronized population of nematodes hatched.

### Experimental procedures

#### Survival assay

Survival experiments of wildtype *C. elegans* strain N2 in 96-well microtiter plates were performed as previously described^39^ with some alterations: since nematodes grow faster at higher temperatures^49^, we performed our survival experiments at 25 °C, we omitted the use of antibiotics and antimycotics and we tested our samples with a final concentration of 1.00% DMSO as a solubilizing agent of extracts. Briefly, 5–18 age-synchronized L1 nematodes were transferred by pipetting to 96-well microtiter plates with 6 mg/mL air dried OP50 in 120 µl S-complete medium, where they grow for 24 h at 25 °C until all worms reach the L3 stage. 5-Fluorodeoxyuridine (FUdR; 0.12 mM final; Sigma-Aldrich, F0503) is added in order to sterilize the worms and to keep the population synchronized^38,95^. The following day (day 0), test samples dissolved in DMSO were added to the sterilized adult worm culture. Reserpine 30 μM (Sigma-Aldrich, 83580) was used as positive control^59^. Nematodes were oxygenized every three days and 5 µL OP50 (c = 100 mg/mL) were added on day 5 of adulthood to prevent starvation. All assays were performed in parallel triplicates. To prevent evaporation, the outer wells were filled with S-complete medium and plates were sealed with parafilm. Plates were stored at 25 °C under light exclusion.

##### Data analysis survival assay

Raw data of the survival assay was recorded with MS Excel 2013 to keep track of living/dead population per well. Survival curves for each plate were determined based on the percentage of living nematodes per well plotted versus time. For each condition at least 3 wells per trial were used. The time point, when 50% of worms were dead/alive (DT_50_) was determined upon survival curves. Mean DT_50_s are given based on the survival curves obtained from three parallel experiments. The deviation of lifespan in comparison to the vehicle control is given as increase/decrease in percentage [DT_50_ extension/reduction (%)]. For better visualization of the DT_50_ extension/reduction all results were depicted as bar charts (GraphPad Prism 4.03). The data values were reported as the mean ± SD. In order to determine whether the differences between control and treated groups were statistically significant an ANOVA (analysis of variance) with Dunnett’s post-test was performed. Significant activity is based on *p* < 0.05.

#### Nile red assay

Synchronized SS104 L1 worms were put on fresh agar plates and kept overnight at 16 °C, then transferred to 25 °C. Experiments were started at L4 stage. 3 to 10 worms were put into the wells of a 96-well plate in S-medium containing 10 mg/mL washed and air dried OP50 bacteria and 100 nM Nile red. Vehicle control and test samples were added to reach a final concentration of 1% DMSO. To prevent evaporation the outer wells were filled with S-complete medium and plates were sealed with parafilm. Worms were kept under light exclusion at 25 °C for 5 days. Worms were paralyzed for image acquisition with NaN_3_.

#### Image acquisition and processing

A Zeiss Z1 Axio Observer inverted fluorescence microscope equipped with a Rhodamine filter and an Axio Cam MRm system was used for imaging. Every living worm was recorded using the same settings and saved as tiff-image in RGB format. The open source software ImageJ^96^ was used for image processing and quantification of fluorescent units. At first, the tiff-images were converted into 8-bit grey scale images and subsequently brightness and contrast adjusted to a mean of 1.2 and a standard deviation of 6.6. For segmentation of the image, the plug-in “Trainable Weka Segmentation”^97^ was used to classify every pixel of the image into “worm” or “background”. Prior performing the classification in ImageJ, the machine learning algorithm *J48 pruned tree classifier*^98^ was trained based on the results of an entropy filter as basis for the classification decision. For an improvement of the results and avoidance of false-positive areas like bacteria and artefacts belonging to the class “worm”, all areas smaller than 3,000 pixels were deleted, followed by manual control. Subsequently the improved results of the classification process were multiplied with the normalized versions of the original images. Binarization of the images was performed with the default threshold-function in ImageJ, resulting in an image that can be quantified. Finally, the total area of white pixels per image were assigned to the fluorescent units of a single worm.

##### Data analysis of the Nile red assay

Mean fluorescent units of worms that have been treated with one condition, repeated in 5–6 replicates, were calculated. The mean fluorescence intensity of vehicle treated control animals was set to 100% and values were expressed as % of control. The presented values show the mean of at least three independent biological experiments. GraphPad Prism 4.03 software was used for all statistical analyses. In order to determine whether the differences between control and treated groups were statistically significant an ANOVA (analysis of variance) with Bonferroni post-test was performed.

## Supplementary information

Supplementary information
