# Supplementary material for: A robust and miniaturized screening platform to study natural products affecting metabolism and survival in Caenorhabditis elegans
Source: Sci Rep. 2020 Jul 23;10:12323. doi: 10.1038/s41598-020-69186-6 (PMC7378205; doi:10.1038/s41598-020-69186-6)
Supplement: Supplementary file 1 — Supplementary information [file 41598_2020_69186_MOESM1_ESM.pdf]

Supplementary Information

A robust and miniaturized screening platform to study natural products affecting metabolism and survival in *Caenorhabditis elegans*

Julia Zwirchmayr[1ǂ], Benjamin Kirchweger[1ǂ], Theresa Lehner[1], Ammar Tahir[1], Dagmar Pretsch[1], Judith M. Rollinger[1*]

Affiliations

[1] Department of Pharmacognosy, Faculty of Life Sciences, University of Vienna, Althanstraße 14, 1090, Vienna, Austria.

[ǂ]these authors contributed equally to this work

Correspondence

Univ.-Prof. Mag. pharm. Dr. Judith M. Rollinger

Department of Pharmacognosy
Faculty of Life Sciences
University of Vienna
Althanstrasse 14, 1090 Vienna

Austria

Phone: +43 1 4277 55255

Fax: +43 1 4277 855255

judith.rollinger@univie.ac.at

Table S1

Table S1 Literature data on 24 NPs with corresponding results from survival and Nile red assay

Organ and Species	Traditional Use, *in vitro* and/or *in vivo* Studies	c [µg/mL]	Survival Assay		Nile Red Assay
			Mean DT_{50} ± SD	DT_{50} Extension/ Reduction [%]	Fluorescence (% of Control)
Herbs of *Andrographis paniculata* (King of Bitters)	South Asian and Southeast Asian medicine; medicinal systems (Ayurveda and Unani); treatment of diabetes, dysentery, fever and malaria[1]	25	17.00 ± 1.73	4.08	246.7 ± 69.5
		100	18.00 ± 1.73	10.20	36850 ± 144.4
Fruits of *Azadirachta indica* (Neem fruits)	Medicinal systems (Ayurveda[2,3], Unani & Siddha[4]) and African pharmacopeia; against malaria[5], piles, urinary disorders, epistaxis, diabetes and for the treatment of wounds[6]	25	15.33 ± 1.26	12.20	114.1 ± 18.0
		100	14.33 ± 5.77	4.88	140.4 ± 45.0
Flowers of *Calendula officinalis* (Pot marigold)	European, Chinese, American and Indian medicine; treatment of wounds[7];	25	17.33 ± 2.31	-0.95	90.5 ± 5.5
		100	17.83 ± 0.29	1.90	102.0 ± 8.9
Lichens of *Cetraria islandica* (Iceland moss)	Throughout the world as traditional medicinal ailment; treatment of tuberculosis, throat irritation and asthma[8,9]; used to promote general health[9,10]	25	18.25 ± 0.35	-30.48	97.2 ± 11.2
		100	16.67 ± 0 76	-4.76	113.2 ± 20.53
Roots of *Cynanchum paniculatum* (Xuchangqing)	TCM; *Cynanchum* sp. are inter alia used to treat rheumatoid arthritis and cancer[11,12]	25	18.33 ± 0.58	**12.24***	165.0 ± 71.0
		100	17.00 ± 1.00	4.08	239.4 ± 187.9
Rhizomes of *Cynanchum stauntonii* (Baiqian)		25	18.17 ± 1.89	11.22	158.0 ± 88.0
		100	19.17 ± 0.76	**17.35***	207.6 ± 118.7
Rhizomes of *Drynaria fortunei* (Guisuibu)	TCM; Korean and Japanese Medicine[13,14]; kidney-tonifying agent, to strengthen bones[13], prevention of osteoporosis and ageing-associated syndromes; radical scavenging activity and protective effect against 6-hydroxydopamine-induced neuronal damage *in vitro*[13]	25	18.17 ± 1.04	11.22	143.1 ± 47.2
		100	18.00 ± 1.80	10.20	119.1± 40.0
Leaves of *Eriobotrya japoinica* (Loquat)	TCM; treatment of inflammation and arthritis[15,16]; cardioprotective effect through attenuation of cardiac hypertrophy in hypertensive rats[17]	25	17.00 ± 0.71	**24.39***	728.1 ± 57.3
		100	22.00 ± 0.00	**34.69***	504.4 ± 143.7
Herbs of *Euphrasia officinalis* (Eyebright herb)	Anthroposophical medicine[18]; conjunctivitis, ophthalmia & ocular allergies[19]	25	16.33 ± 0.58	**19.51****	83.21 ± 4.1
		100	18.17 ± 1.04	**32.93****	114.5 ± 6.8

Organ and Species	Traditional Use, *in vitro* and/or *in vivo* Studies	c [µg/mL]	Survival Assay		Nile Red Assay
			Mean DT_{50} ± SD	DT_{50} Extension/ Reduction [%]	Fluorescence (% of Control)
Fruit bodies of *Fomitopsis pinicola* (Red banded polypore)	Traditional Asian[20] & European medicine[21]; treatment of nausea, headache & liver problems[21]	25	16.33 ± 1.53	-6.67	512.2 ± 279.8
		100	16.50 ± 2.18	-5.71	326.1 ± 163.5
Fruit bodies of *Ganoderma lucidum* (Reishi, língzhī)	TCM for promoting health and longevity[21,22]	25	16.33 ± 1.16	-6.67	180.9 ± 30.5
		100	17.67 ± 1.04	0.95	224.4 ± 10.7
Fruits of *Gardenia jasminoides* (Zhi Zi)	TCM; treatment of inflammation, jaundice, fever, hepatic disorders and hypertension[23-25]; isolates showed hypoglycaemic, hypolipidemic and anti-inflammatory activity *in vivo*[26-29]	25	18.75 ± 0.90	7.18	85.7 ± 2.5
		100	23.83 ± 2.02	**36.84***	**50.3 ± 10.0*****
Fruit bodies of *Gloeophyllum odoratum* (Anise mazegill)	Central Europe, Asia & North America[30]	25	15.83 ± 2.02	15.85	108.4 ± 10.5
		100	17.33 ± 0.76	**26.83****	238.4 ± 31.4
Roots/rhizomes of *Imperata cylindrica* (Alang-Alang)	Traditionally used against nephritis, fever, hypertension[31] and enhancement of immune system[32,33]; anti-hypertensive activity *in vivo*[34]	25	18.50 ± 1.73	5.71	**76.6 ± 5.2***
		100	20.67 ± 1.76	18.10	**74.8 ± 18.3***
Sclerotia of *Inonotus obliquus* (Chaga)	Russia, Poland and Baltic countries; ailment against diabetes, cardiovascular disease and gastrointestinal cancer[35-37]; *I. obliquus* shows hypoglycaemic, anti-inflammatory and anti-lipid peroxidative effects *in vivo*[38-42]	25	13.67 ± 2.02	**33.33***	**75.4 ± 10.1****
		100	14.67 ± 1.04	**43.09****	**23.7 ± 13.3*****
Roots and rhizomes of *Peucedanum ostruthium* (Masterwort)	Austrian & Italian folk medicine; gastro-intestinal, cardiovascular & respiratory diseases[43-46]	25	5.55 ± 0.50	**-46.77****	107.8 ± 39.44
		100	4.17 ± 0.29	**-59.68****	nematotoxic
Herbs of *Scutellaria barbata* (Ban Zhi Lian)	TCM; increased blood circulation and diuresis; treatment of edema[47]; traumatic injuries and tumours[48]	25	21.67 ± 2.26	23.81	**74.7 ± 17.7***
		100	21.83 ± 2.02	24.76	79.5 ± 17.4
Herbs of *Sida cordifolia* (Indian mallow)	Indian, Chinese, African & Brazilian medicine; skin diseases[49,50], inflammation of oral mucosa, nasal congestion, asthmatic bronchitis & rheumatism[51]	25	16. 00 ± 1.80	17.07	322.1± 106.3
		100	15.17 ± 1.04	10.98	211.7 ± 112.7
Flowers/buds of *Syzygium aromaticum* (Cloves)	Tibetan medicine[52]*; in vivo and in vitro* data on beneficial effects against hyperlipidaemia and hyperglycaemia[52-54]	25	17.17 ± 2.26	-1.90	98.1 ± 20.0
		100	19.33 ± 0.76	**10.48***	117.7 ± 21.5

Organ and Species	Traditional Use, *in vitro* and/or *in vivo* Studies	c [µg/mL]	Survival Assay		Nile Red Assay
			Mean DT_{50} ± SD	DT_{50} Extension/ Reduction [%]	Fluorescence (% of Control)
Fruits of *Pimenta dioica* (Allspice)	Tibetan[52], Jamaican[56] & Costa Rican medicine; leaves against menopausal symptoms, dysmenorrhea & dyspepsia[57]; *in vitro* data on beneficial effects against hyperglycaemia[54]	25	12.17 ± 1.89	17.74	197.2 ± 107.
		100	12.83 ± 0.29	**24.19****	248.7 ± 263.4
Fruit bodies of *Piptoporus betulinus* (Birch polypore)	Central European folk medicine[21]; fatigue & immune-enhancing properties[58]	25	16.83 ± 0.29	-3.81	181.8 ± 69.93
		100	16.33 ± 2.02	-6.67	275.1 ± 152.7
Herbs of *Potentilla aurea* (Goldfinger herb)	Southern & Central Europe[59,60]; homeopathic medications; diarrhea, diabetes mellitus & inflammations[60]	25	15.17 ± 2.02	10.98	261.9 ± 8.1
		100	13.67 ± 2.47	0.00	229.6 ± 96.3
Fruits of *Terminalia chebula* (Black Myroblan)	Indian and Iranian traditional medicine[61]; medicinal systems (Ayurveda and Unani); treatment of geriatric diseases, cancer, cardiovascular diseases[62,63] and diabetes[61]; hypolipidemic activity *in vivo*[64]	25	11.67 ± 3.18	12.90	**84.1 ± 7.0****
		100	11.67 ± 3.18	12.90	92.3 ± 5.4
Roots of *Valeriana officinalis* (Valerian)	Europe and parts of Asia; treatment of anxiety, insomnia and psychological stress conditions; *in vitro* promotion of adipocyte differentiation and adiponectin production[65]	25	16.00 ± 0.87	-8.57	92.4 ± 4.8
		100	16.83 ± 1.04	-3.81	**71.8 ± 9.3****

Figure S1

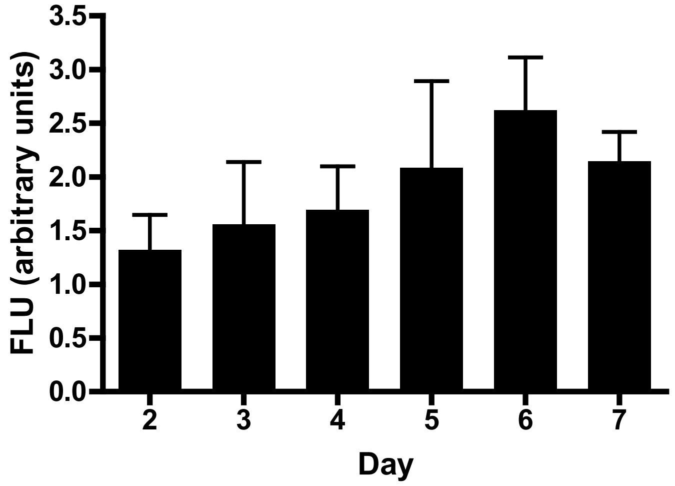

Figure S1 Time dependent Nile red fluorescence of SS104 worms. Worms were incubated with 100 nM Nile red for a defined number of days and their fluorescence was measured by fluorescence imaging as described in Methods. Bar charts represent the mean Nile red fluorescence of two independent experiments expressed as arbitrary fluorescence values (± SD).

Table S2

Table S2 Survival analysis upon treatment with various DMSO concentrations *N* is the total number of worms assayed for survival. One way ANOVA with Dunnett's post-test was used for statistical evaluation. *P*-value < 0.05 was considered as statistically significant.

		Mean DT_{50} ± SD	*N*	DT_{50} extension (%)	*p*-value
	0.00%	15.33 ± 1.55	79	-	
	0.20%	15.83 ± 1.65	88	3.26	ns
DMSO	0.33%	15.17 ± 0.94	87	-1.09	ns
	0.60%	17.83 ± 1.65	80	16.30	ns
	1.00%	17.50 ± 0.82	96	14.13	ns

Table S3

Table S3 Survival analysis upon treatment with mianserine, reserpine and caffeine in comparison to the vehicle control (containing 1% DMSO). *N* is the total number of worms assayed for survival. One way ANOVA with Dunnett's post-test was used for statistical evaluation. *P*-value < 0.05 was considered as statistically significant.

		Mean DT_{50} ± SD	*N*	DT_{50} extension (%)	*p*-value
Control		17.50 ± 1.00	96	-	
Mianserine	50 µM	17.33 ± 2.25	113	11.43	ns
Reserpine	30 µM	23.00 ± 1.50	83	31.43	$p < 0.05$
Caffeine	50 µM	20.50 ± 2.18	78	17.14	ns

Protocol for the preparation of optimized small-scale extracts

as described in Kratz, et al.[66], adapted from Camp, et al.[67].

i. **Extraction**

1. Dried, ground material (300 mg) placed into glass tube (i.e. centrifugation tube)
2. Defatting with n-hexane
   → 5 ml, shake for ~ 1 min
3. Centrifugation (10 min, 3.500 rpm)
   → discard extract and repeat step 2. and 3.
4. Extraction with CH_2Cl_2
   → 7 ml; 15 min ultrasonic bath
5. Filter extract trough pipette stowed with cotton wool
   → extract transferred into a round-bottom flask
6. Second extraction of same natural material with MeOH
   → 13 ml; 15 min ultrasonic bath
7. Centrifuge (10 min, 3.500 rpm)
   → combine and dry MeOH and CH_2Cl_2 extracts on e.g. rotary evaporator

ii. Removal of tannins

8. Reconstitution of dried extract in MeOH (4 ml)
9. Loading extract onto SPE cartridge
   → unpacked 3 ml SPE cartridges with 20 µm pore size frit (Phenomenex)
   → fill with 900 mg polyamide gel (CC-6)
10. Wash loaded solid phase gel 2 times with MeOH (2 x 4 ml)
11. Reduce the volume of the combined MeOH eluates e.g. rotary evaporator
   → transfer to pre-labeled and pre-weighted tubes
   → final drying step (desiccator)

Figure S2

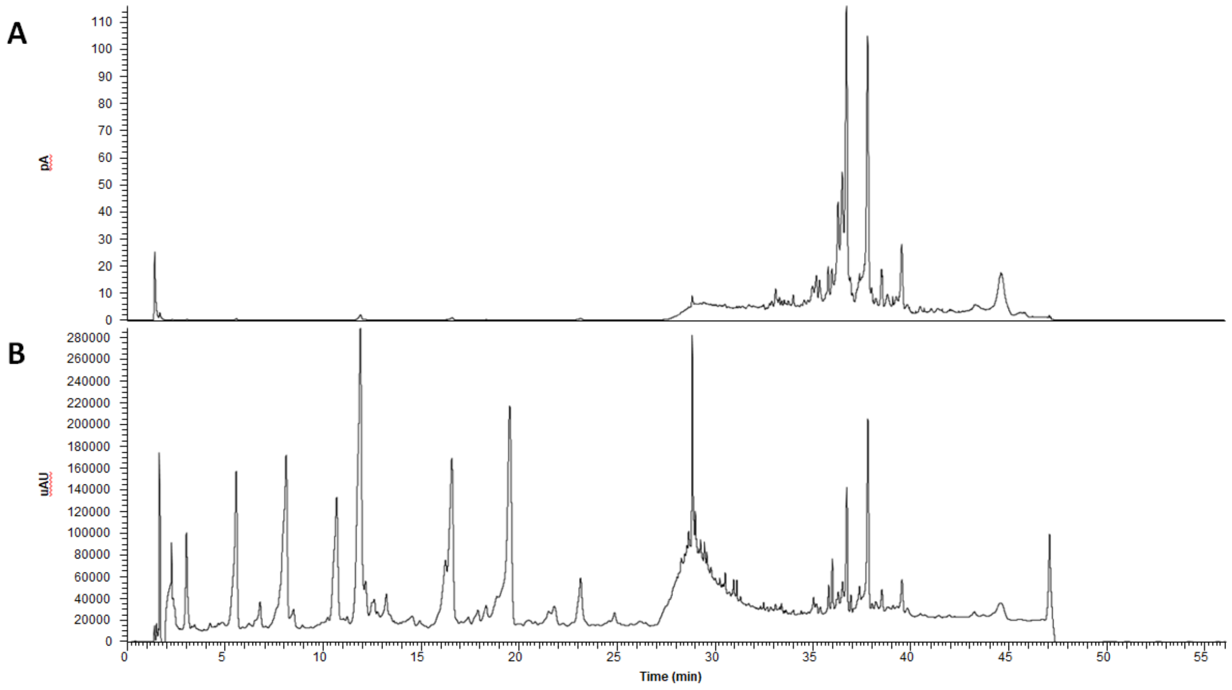

Figure S2 HPLC chromatogram of *I. obliquus* sclerotia extract (**A**) CAD chromatogram and (**B**) PDA full range-scan

Figure S3

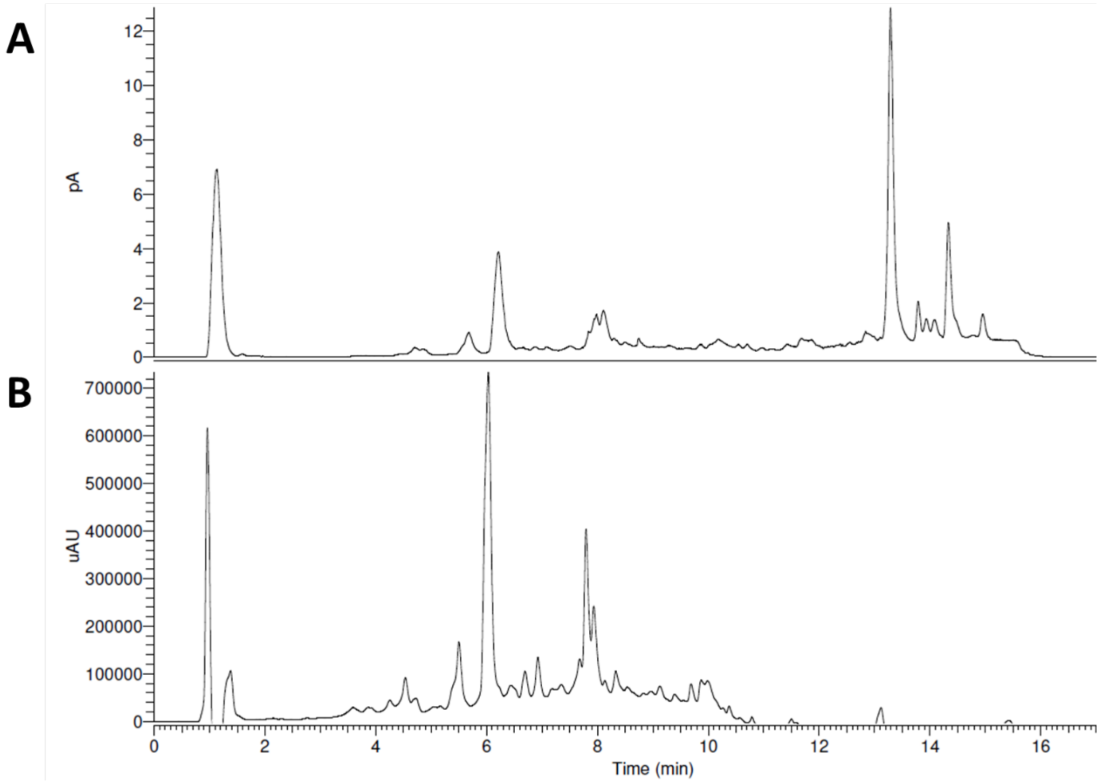

Figure S3 HPLC chromatogram of *G. jasminoides* fruit extract (**A**) CAD chromatogram and (**B**) PDA full range-scan

HPLC chromatograms of *I. obliquus* and *G. jasminoides* extracts The extracts of *I. obliquus* sclerotia and *G. jasminoides* fruits were chromatographed over a Dionex Ultimate 3000 RS system, equipped with a photo diode array (PDA) detector and a charged aerosol detector (CAD). The sclerotia extract of *I. obliquus* (**Figure S2**) was chromatographed over an Agilent Zorbax SB-C18 column (3.5 µm, 4.6 x 150 mm). The mobile phase consisted of H2O+0.1% formic acid/MeCN+0.1% formic acid. The gradient was: 0 min 5%/95%, 35 min 98%/2%, 40 min 98%/2%, 41 min 5%/95%, 46 min 5%/95%. Conditions: column temperature, 40°C; flow rate, 1 mL/min; injection volume, 10 µL; detection wavelengths, 210 and 360 nm and PDA full range scan 190-400 nm. HPLC analysis of *G. jasminoides* fruit extract (**Figure S3**) was performed on a Waters Acquity UPLC HSS T3 column (1.8 µm, 2.1 x 100 mm). The mobile phase consisted of MeCN+0.1% formic acid/H2O+0.1% formic acid gradient (0 min 5%/95%, 0.50 min 5%/95%, 8.00 min 45%/55%, 8.10 min 98%/2%, 13.00 min 98%/2%, 13.10 min 5%/95% and 18.00 min 5%/95%). Conditions: column temperature, 40°C; flow rate, 0.3 mL/min; injection volume, 1 µL; detection wavelengths, 210 and 360 nm and PDA full range scan 190-400 nm.

References Supplementary Information

1 Hossain, M. S., Urbi, Z., Sule, A. & Hafizur Rahman, K. M. *Andrographis paniculata* (Burm. f.) Wall. ex Nees: A review of ethnobotany, phytochemistry, and pharmacology. *Sci. World J.* **2014**, 274905-274905 (2014).

2 Subapriya, R. & Nagini, S. Medicinal properties of neem leaves: a review. *Anti-Cancer Agents Med. Chem.* **5**, 149-156 (2005).

3 Xiang, X., Wu, L., Mao, L. & Liu, Y. Antioxidative and antiapoptotic neuroprotective effects of *Azadirachta indica* in Parkinson induced functional damage. *Mol. Med. Rep.* **17**, 7959-7965 (2018).

4 Patel, S. M., Nagulapalli Venkata, K. C., Bhattacharyya, P., Sethi, G. & Bishayee, A. Potential of neem (*Azadirachta indica* L.) for prevention and treatment of oncologic diseases. *Semin. Cancer Biol.* **40-41**, 100-115 (2016).

5 Chianese, G. *et al.* Antiplasmodial triterpenoids from the fruits of neem, *Azadirachta indica*. *J. Nat. Prod.* **73**, 1448-1452 (2010).

6 Paul, R., Prasad, M. & Sah, N. K. Anticancer biology of *Azadirachta indica* L (neem): A mini review. *Cancer Biol. Ther.* **12**, 467-476 (2011).

7 Givol, O. *et al.* A systematic review of *Calendula officinalis* extract for wound healing. *Wound Repair Regen.* **27**, 548-561 (2019).

8 Surayot, U. *et al.* Structural characterization of a polysaccharide from *Certaria islandica* and assessment of immunostimulatory activity. *Process Biochem.* **83**, 214-221 (2019).

9 Freysdottir, J., Omarsdottir, S., Ingólfsdóttir, K., Vikingsson, A. & Olafsdottir, E. S. *In vitro* and *in vivo* immunomodulating effects of traditionally prepared extract and purified compounds from *Cetraria islandica*. *Int. Immunopharmacol.* **8**, 423-430 (2008).

10 Ingólfsdóttir, K. in *Bioactive Carbohydrate Polymers. Proceedings of the Phythochemical Society of Europe* Vol. 44 (ed B.S. Paulsen) Ch. 3, 25-36 (Springer, 2000).

11 Han, L. *et al.* Ethnobotany, phytochemistry and pharmacological effects of plants in genus *Cynanchum* Linn. (Asclepiadaceae). *Molecules.* **23**, 1194 (2018).

12 Lee, S. K., Nam, K. A. & Heo, Y. H. Cytotoxic activity and G2/M cell cycle arrest mediated by antofine, a phenanthroindolizidine alkaloid isolated from *Cynanchum paniculatum*. *Planta Med.* **69**, 21-25 (2003).

13 Kuo, H.-C. et al. Protective effects of *Drynaria fortunei* against 6-hydroxydopamine-induced oxidative damage in B35 cells via the PI3K/AKT pathway. *Food Funct.* **5**, 1956-1965 (2014).

14 Yang, Z.-Y., Kuboyama, T., Kazuma, K., Konno, K. & Tohda, C. Active constituents from *Drynaria fortunei* rhizomes on the attenuation of Aβ25–35-induced axonal atrophy. *J. Nat. Prod.* **78**, 2297-2300 (2015).

15 Kuraoka-Oliveira, A. M. *et al.* Anti-inflammatory and anti-arthritic activity in extract from the leaves of *Eriobotrya japonica*. *J. Ethnopharmacol.* **249**, 112418 (2020).

16 Cha, D. S., Eun, J. S. & Jeon, H. Anti-inflammatory and antinociceptive properties of the leaves of *Eriobotrya japonica*. *J. Ethnopharmacol.* **134**, 305-312 (2011).

17 Chiang, J.-T. et al. *Eriobotrya japonica* ameliorates cardiac hypertrophy in H9c2 cardiomyoblast and in spontaneously hypertensive rats. *Environ. Toxicol.* **33**, 1113-1122 (2018).

18 Stoss, M., Michels, C., Peter, E., Beutke, R. & Gorter, R. W. Prospective cohort trial of *Euphrasia single*-dose eye drops in conjunctivitis. *J. Altern. Complement. Med.* **6**, 499-508 (2000).

19 Liu, Y. et al. Protective effects of *Euphrasia officinalis* extract against ultraviolet B-induced photoaging in normal human dermal fibroblasts. *Int. J. Mol. Sci.* **19**, 3327 (2018).

20 Hao, L., Sheng, Z., Lu, J., Tao, R. & Jia, S. Characterization and antioxidant activities of extracellular and intracellular polysaccharides from *Fomitopsis pinicola*. *Carbohydr. Polym.* **141**, 54-59 (2016).

21 Grienke, U., Zoll, M., Peintner, U. & Rollinger, J. M. European medicinal polypores--a modern view on traditional uses. *J. Ethnopharmacol*. **154**, 564-583 (2014).

22 Wang, J., Cao, B., Zhao, H. & Feng, J. Emerging roles of *Ganoderma lucidum* in anti-aging. *Aging Dis.* **8**, 691-707 (2017).

23 Xiao, W., Li, S., Wang, S. & Ho, C.-T. Chemistry and bioactivity of *Gardenia jasminoides. J. Food Drug Anal.* **25,** 43-61 (2017).
24 Chinese Pharmacopoeia Committee. *The Pharmacopoeia of the People's Republic of China* (ed. 2015). (China Medical Science Press, 2015).
25 Koo, H.-J. et al. Anti-inflammatory effects of genipin, an active principle of *Gardenia*. *Eur. J. Pharmacol.* **495**, 201-208 (2004).
26 Wu, S.-Y. *et al.* Effect of geniposide, a hypoglycemic glucoside, on hepatic regulating enzymes in diabetic mice induced by a high-fat diet and streptozotocin. *Acta Pharmacol. Sin.* **30,** 202-208 (2009).
27 Sheng, L., Qian, Z., Zheng, S. & Xi, L. Mechanism of hypolipidemic effect of crocin in rats: crocin inhibits pancreatic lipase. *Eur. J. Pharmacol.* **543,** 116-122 (2006).
28 He, S. Y. *et al.* Effect of crocin on experimental atherosclerosis in quails and its mechanisms. *Life Sci.* **77,** 907-921 (2005).
29 Guan, L. *et al.* Genipin ameliorates age-related insulin resistance through inhibiting hepatic oxidative stress and mitochondrial dysfunction. *Exp. Gerontol.* **48,** 1387-1394 (2013).
30 Grienke, U. et al. Lanostane triterpenes from *Gloeophyllum odoratum* and their anti-influenza effects. *Planta Med.* **85**, 195-202 (2019).
31 Anggraeni, N., Syamsunarno, M. R. A. A., Widyastuti, R., Puspitasari, I. M. & Praptama, S. Potential dual effect anti-inflammatory and anti-platelet of cogon grass ethanol extract on diabetic mice a preliminary study. *J. Phys.: Conf. Ser.* **1246**, 012006 (2019).
32 Wang, Y., Shen, J. Z., Chan, Y. W. & Ho, W. S. Identification and growth inhibitory activity of the chemical constituents from *Imperata cylindrica* aerial part ethyl acetate extract. *Molecules.* **23**, 1807 (2018).
33 Pinilla, V. & Luu, B. Isolation and partial characterization of immunostimulating polysaccharides from *Imperata cylindrica*. *Planta Med.* **65**, 549-552 (1999).
34 Ruslin et al. Anti-hypertensive activity of alang - alang (*Imperata cylindrica* (L.) Beauv. root methanolic extract on male Wistar rat. *Int. J. Res. Pharm. Sci.* **4**, 537-542 (2013).
35 Duru, K. C., Kovaleva, E. G., Danilova, I. G. & van der Bijl, P. The pharmacological potential and possible molecular mechanisms of action of Inonotus obliquus from preclinical studies. Phytother. Res. **33,** 1966-1980 (2019).
36 Shikov, A. N. *et al.* Medicinal plants of the Russian pharmacopoeia; their history and applications. *J. Ethnopharmacol.* **154,** 481-536 (2014).
37 Park, Y. M. et al. *In vivo* and *in vitro* anti-inflammatory and anti-nociceptive effects of the methanol extract of *Inonotus obliquus*. *J. Ethnopharmacol.* **101**, 120-128 (2005).
38 Wu, T. *et al.* Ameliorating effects of *Inonotus obliquus* on high fat diet-induced obese rats. *Acta Biochim. Biophys. Sin.* **47,** 755-757 (2015).
39 Na, H. G. *et al.* Secondary fermented extract of chaga-cheonggukjang attenuates the effects of obesity and suppresses inflammatory response in the liver and spleen of high-fat diet-induced obese mice. *J. Microbiol. Biotechnol.* **29,** 739-748 (2019).
40 Sun, J. E. *et al.* Antihyperglycemic and antilipidperoxidative effects of dry matter of culture broth of *Inonotus obliquus* in submerged culture on normal and alloxan-diabetes mice. *J. Ethnopharmacol.* **118,** 7-13 (2008).
41 Lu, X., Chen, H., Dong, P., Fu, L. & Zhang, X. Phytochemical characteristics and hypoglycaemic activity of fraction from mushroom *Inonotus obliquus*. *J. Sci. Food Agric.* **90,** 276-280 (2010).
42 Wang, J. et al. Antidiabetic activities of polysaccharides separated from *Inonotus obliquus* via the modulation of oxidative stress in mice with streptozotocin-induced diabetes. *PLoS One.* **12**, e0180476 (2017).
43 Vogl, S. et al. Identification and quantification of coumarins in *Peucedanum ostruthium* (L.) Koch by HPLC-DAD and HPLC-DAD-MS. *J. Agric. Food Chem.* **59**, 4371-4377 (2011).
44 Palmioli, A. et al. bioNMR-based identification of natural anti-Aβ compounds in *Peucedanum ostruthium. Bioorg. Chem.* **83**, 76-86 (2019).

45 Vogl, S. et al. Ethnopharmacological *in vitro* studies on Austria's folk medicine - an unexplored lore *in vitro* anti-inflammatory activities of 71 Austrian traditional herbal drugs. *J Ethnopharmacol*. **149**, 750-771 (2013).
46 Hiermann, A. & Schantl, D. Antiphlogistic and antipyretic activity of *Peucedanum ostruthium. Planta Med.* **64**, 400-403 (1998).
47 Qiong, C., Khalid, R., Su-Juan, W., Shuang, Z. & Hong, Z. *Scutellaria barbata*: a review on chemical constituents, pharmacological activities and clinical applications. *Curr. Pharm. Des.* **26**, 160-175 (2020).
48 Gao, J., Yin, W. & Corcoran, O. From *Scutellaria barbata* to BZL101 in cancer patients: phytochemistry, pharmacology, and clinical evidence. *Nat. Prod. Commun.* **14**, 1934578X19880645 (2019).
49 Bonjardim, L. R. et al. *Sida cordifolia* leaf extract reduces the orofacial nociceptive response in mice. *Phytother. Res*. 25, 1236-1241 (2011).
50 Kumar, S., Lakshmi, P. K., Sahi, C. & Pawar, R. S. *Sida cordifolia* accelerates wound healing process delayed by dexamethasone in rats: effect on ROS and probable mechanism of action. *J. Ethnopharmacol.* **235**, 279-292 (2019).
51 Rejitha, S., Prathibha, P. & Indira, M. Amelioration of alcohol-induced hepatotoxicity by the administration of ethanolic extract of *Sida cordifolia* Linn. *Br. J. Nutr.* **108**, 1256-1263 (2012).
52 Brunner-La Rocca, H. P., Schindler, R., Schlumpf, M., Saller, R. & Suter, M. Effects of the Tibetan herbal preparation PADMA 28 on blood lipids and lipid oxidisability in subjects with mild hypercholesterolaemia. *VASA.* **34**, 11-17 (2005).
53 Khathi, A., Serumula, M. R., Myburg, R. B., Van Heerden, F. R. & Musabayane, C. T. Effects of *Syzygium aromaticum*-derived triterpenes on postprandial blood glucose in streptozotocin-induced diabetic rats following carbohydrate challenge. *PloS One.* **8**, e81632-e81632 (2013).
54 Ladurner, A. et al. Allspice and clove as source of triterpene acids activating the G protein-coupled bile acid receptor TGR5. *Front. Pharmacol.* **8**, 468 (2017).
56 Padmakumari, K. P., Sasidharan, I. & Sreekumar, M. M. Composition and antioxidant activity of essential oil of pimento (*Pimenta dioica* (L) Merr.) from Jamaica. *Nat. Prod. Res.* **25**, 152-160 (2011).
57 Doyle, B. J. et al. Isolation and identification of three new chromones from the leaves of *Pimenta dioica* with cytotoxic, oestrogenic and anti-oestrogenic effects. *Pharm Biol.* **56**, 235-244 (2018).
58 Kamo, T., Asanoma, M., Shibata, H. & Hirota, M. Anti-inflammatory lanostane-type triterpene acids from *Piptoporus betulinus*. *J. Nat. Prod.* **66**, 1104-1106 (2003).
59 Muhlemann, H. Falsification of herba Potentillae aureae L. *Pharm. Acta Helv.* **13**, 67-70 (1938).
60 Tomczyk, M. & Latté, K. P. *Potentilla*—A review of its phytochemical and pharmacological profile. *J. Ethnopharmacol.* **122**, 184-204 (2009).
61 Jokar, A., Masoomi, F., Sadeghpour, O., Nassiri-Toosi, M. & Hamedi, S. Potential therapeutic applications for *Terminalia chebula* in Iranian traditional medicine. *J. Tradit. Chin. Med.* **36**, 250-254 (2016).
62 Afshari, A. R., Sadeghnia, H. R. & Mollazadeh, H. A review on potential mechanisms of *Terminalia chebula* in Alzheimer's disease. *Adv. Pharmacol. Sci*. **2016**, 8964849-8964849 (2016).
63 Saleem, A., Husheem, M., Harkonen, P. & Pihlaja, K. Inhibition of cancer cell growth by crude extract and the phenolics of *Terminalia chebula* Retz. fruit. *J. Ethnopharmacol.* **81**, 327-336 (2002).
64 Maruthappan, V. & Shree, K. S. Hypolipidemic activity of Haritaki (*Terminalia chebula*) in atherogenic diet induced hyperlipidemic rats. *J. Adv. Pharm. Technol. Res.* **1**, 229-235 (2010).
65 Harada, K. et al. The effect of methanolic *Valeriana officinalis* root extract on adipocyte differentiation and adiponectin production in 3T3-L1 adipocytes. *Plant Foods Hum. Nutr*. **75**, 103-109 (2020).
66 Kratz, J. M. et al. hERG channel blocking ipecac alkaloids identified by combined *in silico - in vitro* screening. *Planta Med.* **82**, 1009-1015 (2016).
67 Camp, D., Davis, R. A., Campitelli, M., Ebdon, J. & Quinn, R. J. Drug-like properties: guiding principles for the design of natural product libraries. *J Nat Prod.* **75**, 72-81, (2012).
